# Jared Diamond Confidential *Reflections on Writing, Science, and Life*

**DOI:** 10.4269/ajtmh.17-0085

**Published:** 2017-04-05

**Authors:** Claire Panosian Dunavan

**Affiliations:** 1 University of California, Los Angeles, California. E-mail: cpanosian@mednet.ucla.edu

Sometime after a trip to New Guinea in 1972, perhaps the world's most noted ornithologist-physiologist-turned-evolutionary biologist and biogeographer set about “the modest task of trying to explain the broad pattern of human history, on all the continents, for the last 13,000 years.” In 1998, *Guns, Germs, and Steel* won the Pulitzer Prize.

The book's thesis, in short, is that specific geographic factors tipped the balance at the dawn of civilization. In Eurasia, which was blessed with many plants and animals suitable for domestication, geography fueled the rise of agriculture, which in turn allowed human communities to grow and differentiate into leaders, workers, and inventors. Exposure to domesticated animals' germs also buttressed Eurasians' defenses against potentially lethal infections. For these reasons, Diamond asserts, the people of the Fertile Crescent and their descendants more rapidly advanced and ultimately conquered the indigenous people of Africa, the New World, Australia, and the South Pacific. At the same time it explains global disparities, *Guns, Germs, and Steel* also refutes the notion that any given race is biologically or intellectually superior to another.

In honor of this year's 20th anniversary re-release of his famous work, the *American Journal of Tropical Medicine and Hygiene* interviewed long-time University of California Los Angeles professor Jared Diamond. What follows is vintage, free-range Diamond, first captured on tape in November 2016.

***Guns, Germs, and Steel* took a long time to conceive and write. Please share the book's back-story and one or two “Aha!” moments.** The central question hit me as soon as I first went to New Guinea in 1964 with the naïve idea that I would be meeting “primitive” people. It took me about a day to realize, yeah, they had stone tools, but in no respect were they themselves primitive. That raised the question: “Why are they [and not us] the ones who ended up with stone tools?” The question was nudged in 1972 when I was walking around a New Guinea offshore island and was overtaken by a guy called Yali, who chatted, chatted, chatted, and then, after an hour or so, turned and looked at me and said: “So why is it that you white people ended up with all the cargo?” I blabbered out something, but as soon as I said it I knew that my explanation was wrong.

The next step was when I began writing articles for the public for *Discover* and *Natural History* magazines. One of those articles—I think it was called “The First Conquerors”—basically set out the thesis. It asked the question why Europeans conquered Native Americans rather than vice versa. That's when I realized that this was something that I wanted to write a book about.

But you asked about “Eureka” moments. There were actually two. The first occurred in 1990 when an archaeologist at the University of North Carolina was discussing with me why Europeans conquered Native Americans, and we started talking about agriculture. “Yes, there's corn and maize and tomatoes and wheat and so on,” he said, “but an important consideration is that Europe has an east–west axis whereas the Americas run north–south, and that made it difficult for crops, as well as technology, to spread in the Americas.” A year or two later, when I was at the University of Utah preparing to give several Tanner lectures and had 2 or 3 days to kill, I thought to myself, “I need another lecture topic. Why not see if I could have some original insights into African history?” So I looked at a map of Africa, and it was, “My God, look at that map. Here is another continent with a north–south axis.”

In fact, slow north–south spreads are an issue not only in the New World, with Andean potatoes and llamas never reaching Mexico and Mexican turkeys never reaching Peru, but also in Africa, where the spread of cattle and sheep and goats from the Fertile Crescent was very slow, and where wheat and Mediterranean crops from the north never reached the south at all.

**You've always loved geography. You'd been looking at maps all your life, right?** I was born in 1937, so yes. My father, on the blackboard of my bedroom, had two maps. There was a map of the European theater and a map of the Pacific theater, and each day Dad moved the pins, so I grew up with maps in my face. Then in 1950, Dad took my sister and me and Mum to Europe for the first time, and there was geography again in my face. I've loved it ever since.

**Looking back, what were some of the most surprising reactions to *Guns, Germs, and Steel*?** The biggest surprise for me was the Pulitzer Prize. I didn't know anything about Pulitzer Prizes, so it was not like I was waiting by my phone at 11:59 am on the first Tuesday in April. Instead, I happened to be in my office and I got a call. “Mr. Diamond, is this Mr. Diamond?” “Yeah.” “So what is your reaction to having won the Pulitzer Prize for nonfiction?” My first reaction was “My God!” and my second reaction was “How do I know this isn't a hoax?”

As to negative reactions to the book, I never really anticipated them because I found the subject so interesting. It never occurred to me that a substantial number of academics would resist the idea of anything other than culture impacting human civilization. Even now, there are lots of people who will not have their world view changed.

**Since you brought it up, let's move to geographic determinism vis-à-vis modern disparity. We will soon have a new political administration in this country. If you were advising them, what would you say?** Well, I don't think the new administration is going to be receptive to my point of view, but I would make two points. One is that the reason why the United States today is rich and Afghanistan is poor is not that Americans are bright and Afghans are stupid and incapable of anything bold or technologically advanced. Similarly, here in the United States in 1941, it was widely believed that the Japanese were barbarians and incapable of anything intelligent. That mistake was very costly to us because the result was 4 years of war. So my first message would be this. The reason why many countries are poor is not that their inhabitants are unintelligent; there are historical reasons why their inhabitants have not become rich, but at the same time they remain capable of [perpetrating events like] Pearl Harbor or the 2001 attacks. Message number two? In today's globalized world, we are no longer just being generous by giving foreign aid: we're also being selfish because we are thereby helping ourselves. When countries are poor, they can harbor billions of people who are desperate and support terrorists.

It's now in the United States's selfish interest to try to do what we can to increase the standard of living around the world. If we fail to do so, that and climate change will be the two biggest things that will do us in.

**What about emerging infectious diseases? In recent decades, more have arisen in areas where conquerors traditionally imported germs.** There are certainly people who consider emerging disease as one of the big threats for the world. For example, when I first met Bill Gates and we were chatting, he said, “I know that you, Jared, are very concerned about environmental issues, and maybe you're right, but I'm more concerned about the risks of emerging diseases….”

Personally, I don't see them as something that threatens human survival or civilization. Yes, in the past, disease killed most of the inhabitants of the New World, and I suppose it's conceivable that an emerging disease might kill on that scale again, but I'd say the odds are against it because of modern medicine. At the same time, there will always be lots of people, especially rich, educated people, who will complain if certain emerging diseases, like Ebola, become even a relatively tiny problem.

**Let's return to agriculture. What worries you there?** Well, in a worst-case scenario, say a nuclear war in the 2050s that doesn't kill everybody but does destroy First World civilization, who's going to survive? That answer is clear. The people who survive will be New Guineans, because for 7,000 years they've had agriculture that didn't rely on outside inputs. They've also had recent experience of stone tools, whereas probably not a single person who reads your article has the faintest idea how to make a stone tool or how to select a suitable stone. Most of them also wouldn't have any idea how to practice agriculture. Maybe a few have planted gardens, but they still don't know how to grow things on a large scale.

So one of the risks we face if we destroy First World civilization is that we're not in a position to build it back up again. Some people might say: “Okay, if we destroy First World civilization, it will take a few decades before we develop the iron and copper and oil to do that.” But no, we won't build it up, because the resources we exploited previously were immediately accessible on the surface, and we have already removed them using primitive technologies. We'll never recreate First World society again.

**Another hot-button, modern issue involves genetically modified crops.** Yes, there's much reflex opposition to genetically modified (GM) crops. My first response is that everything we eat today is genetically modified because the act of domestication itself introduced change. Initially, GM crops were unintended byproducts of harvesting plants and recovering some seeds. Now we modify intentionally, but the results are still genetic change. Objecting to eating something on the grounds that it is genetically different is nonsense if you don't want to live as a hunter-gatherer.

On the other hand, there are issues such as GM crops' increased resistance to herbicides, which could have negative consequences on insects and earthworms. So, yes, there are some negatives that, it is my impression, have not been adequately or seriously considered by Monsanto and other commercial producers.

Finally, people talk about GM crops as the solution for the world's hunger. Well, Malthus, Malthus, Malthus. Nobody's ever figured out a way of solving the world's hunger while also preventing people who have enough food from using their adequate diets to turn out new babies. This will only make the day of reckoning more painful, because it will come not with 1 or 7 billion people but with 12 billion people, which will be even harder.

**As current and future generations grapple with these challenges, how can we increase their science literacy?** First let me say that this is a special problem here. The United States is, I think, unique among First World countries in the anti-intelligence and anti-science movement. It's not just that the majority of Americans don't believe in evolution. It's that, incredibly, the majority of American college graduates majoring in biology don't believe in evolution, which is not just important for a theoretical understanding of the world, but for practical things like antibiotic resistance and knowing why you may or may not come down with Type 2 diabetes.

As for how to overcome American resistance to grasping science, I don't see any other way except through science writing that appeals to the public. Unfortunately, there are two things weighing against this in the educated science establishment itself. One is academia's suspicion and prejudice against academics who write for the public. The other thing I see in the science establishment is the lack of promotion of scientists writing for the public and the resort to science journalists. With all due respect to journalists, a scientist can do it better. Science journalists regularly make the claim that such-and-such discovery is overthrowing previous knowledge—they standardly have sentences containing phrases like “a new paradigm”—they water it down.

The first way to get scientists motivated to write for the public is to have opportunities in *Nature* and *Science*. But *Nature* and *Science*, and now, worst of all, the *Proceedings of the National Academy of Sciences*, are not encouraging scientists to write for the public. Scientists regularly complain that the government doesn't understand science and, therefore, the government doesn't provide enough funding for NIH or NSF. But the reason for that is entirely the fault of scientists themselves who don't make science vivid for the politicians and, in fact, make life difficult for scientists who *do* want to make science vivid for politicians.

**Speaking of vivid, let's finish on an up-note. How did you hit upon the title for *Guns, Germs, and Steel*?** The credit belongs to Marie [Diamond's wife]. Initially there were various titles for that book, including some three-word titles, but when Marie came up with *Guns, Germs, and Steel*, the virtue was that it was monosyllabic and hammer-like [Diamond makes a noise on table]. Then came the pushback! My wonderful editor at Norton responded: “The sequence is wrong. First comes wheat, which you should mention first, and then come germs, and then comes steel, and then comes guns, so the title should really be ‘Wheat, Germs, Steel, and Guns.’” But ‘Wheat, Germs, Steel, and Guns’ doesn't sound like *Guns, Germs, and Steel*, does it? People remember *Guns, Germs, and Steel*: it sounds like the first four notes of Beethoven's Fifth Symphony. [Diamond laughs]

At the time this interview was conducted, the 38th translation of “Guns, Germs, and Steel” was already in press. Diamond's seventh book, “Crisis and Change,” will be published in 2019.

